# Small Nucleic Acid Therapeutics for Ocular Diseases: Progress, Challenges, and Future Perspectives

**DOI:** 10.3390/pharmaceutics18091189

**Published:** 2026-09-20

**Authors:** Qi Guo, Lushu Chen, Ziyan Wu, Qiuyang Zhang, Jinsong Xue, Huiying Zhang

**Affiliations:** 1 Affiliated Eye Hospital, Nanjing Medical University, 138 Hanzhong Road, Nanjing 210029, China; nukeqiii@stu.njmu.edu.cn (Q.G.); shushu972016@outlook.com (L.C.); wuziyan@stu.njmu.edu.cn (Z.W.); zqyinnj@163.com (Q.Z.); 2 The Fourth School of Clinical Medicine, Nanjing Medical University, 140 Hanzhong Road, Nanjing 210029, China

**Keywords:** small nucleic acid therapeutics, ocular diseases, drug delivery, gene therapy

## Abstract

Ocular diseases remain a major global health challenge with substantial unmet therapeutic needs. Small nucleic acid therapeutics offer a precise strategy to regulate disease-related mRNAs or noncoding RNAs through base pairing, thereby modulating protein expression at the RNA level. Major modalities include antisense oligonucleotides, small interfering RNAs, microRNA-based therapeutics, small activating RNAs, and nucleic acid aptamers, which act through RNA degradation, RNA interference, splicing modulation, microRNA regulation, transcriptional activation, or structure-dependent target binding. These properties make them attractive for ocular diseases involving genetic defects, pathological angiogenesis, inflammation, fibrosis, or neurodegeneration. However, their clinical translation in ophthalmology remains limited by poor molecular stability, insufficient tissue retention, immune activation, off-target effects, and inefficient delivery to target ocular tissues. Rational oligonucleotide design, appropriate local administration routes, and optimized delivery platforms are therefore essential for improving stability, tissue penetration, cellular uptake, and therapeutic durability. This review summarizes the major classes, mechanisms, chemical modification strategies, ocular delivery systems, and therapeutic applications of small nucleic acid drugs in ophthalmology. We also discuss lessons from clinical successes and failures and propose future directions for safe, durable, and individualized ocular therapy.

## 1. Introduction

Visual impairment is a major and growing global health burden. According to the World Health Organization (WHO) World Report on Vision, at least 2.2 billion people worldwide live with a vision impairment, of whom at least 1 billion have a condition that could have been prevented or has yet to be addressed [1]. Most cases are attributable to age-related macular degeneration (AMD), diabetic retinopathy (DR), glaucoma, or inherited retinal disorders such as Leber congenital amaurosis. Each of these diseases progresses through a complex interplay of pathological angiogenesis, neurodegeneration, chronic inflammation, and genetic lesions.

Current treatments, including intravitreal anti-VEGF injections, laser photocoagulation, and vitreoretinal surgery, slow disease progression but rarely address the underlying pathology. Protein and antibody-based drugs have limited intraocular half-lives and therefore require repeated intravitreal injections, which increase the risk of procedure-related complications such as endophthalmitis and add to the overall treatment burden [2,3,4,5]. Conventional therapies also cannot correct genetic defects or meaningfully coordinate multiple disease-relevant pathways. Moreover, ocular barriers, especially the blood–retinal barrier, substantially limit drug penetration into posterior segment tissues and reduce therapeutic access to target cells [6]. Small nucleic acid therapeutics offer an alternative route. They belong to the third wave of biotherapeutics, following small molecules and monoclonal antibodies. Rather than modulating proteins directly, they act at the RNA level through RNA interference (RNAi), antisense-mediated silencing, or CRISPR-Cas9 editing. This approach provides high target specificity, access to previously undruggable targets, durable effects, and the capacity to engage multiple pathways simultaneously [7]. For refractory and inherited eye diseases, these features make nucleic acid drugs particularly attractive.

The field has developed rapidly. In the 1970s, complementary oligonucleotides were first shown to block viral replication, providing an early demonstration of antisense technology [8]. Fomivirsen, the first approved ASO, reached the market in 1998 [9]. The discovery of microRNAs and the elucidation of RNA interference machinery subsequently enabled targeted gene silencing [10]. In 2018, Onpattro became the first siRNA therapeutic delivered by a non-viral platform to receive regulatory approval [11]. Since then, advances in molecular biology, bioinformatics, and nanotechnology have accelerated progress. By the end of 2023, several small nucleic acid therapeutics, including ASOs, siRNAs, and aptamers, had received regulatory approval from the FDA or EMA for rare genetic diseases, chronic disorders, and selected ophthalmic indications [12]. These approvals support the clinical feasibility of small nucleic acid therapeutics, but broader application in ophthalmology still depends on effective ocular delivery. Small nucleic acids must be protected from degradation, retained within target tissues, and delivered to the appropriate ocular cell types. Lipid nanoparticles, polymeric nanocarriers, and extracellular vesicle-based systems have therefore attracted increasing attention, as they may improve molecular protection, tissue retention, and cellular uptake. These advances are accelerating clinical translation and expanding the range of potential ophthalmic applications [13].

This review covers the classification, mechanisms, chemical modifications, and delivery strategies for small nucleic acid therapeutics, with an emphasis on recent clinical progress, therapeutic applications, and the hurdles that continue to limit translation. Because several recent reviews have covered closely related territory—including the RNA-based ocular therapeutic and delivery landscape [14], ocular RNA nanomedicine platforms [15], the general small nucleic acid drug pipeline [16], and inherited retinal disease-focused nucleic acid approaches [17]—we explicitly distinguish our contribution along three axes. First, we provide a modality-level analysis of clinical successes and failures, tiering each therapeutic by regulatory and developmental status rather than presenting all agents at the same evidentiary level. Second, we dedicate full coverage to chemical modification chemistry (backbone, base, and ribose modifications) and to small activating RNAs, which are largely absent from prior ocular reviews. Third, we pair posterior-segment indications with ocular surface disease (dry eye disease) and add a comparative analysis of topical, intravitreal, subretinal, and suprachoroidal delivery routes, which the overlapping reviews address only briefly. Our aim is to provide a practical foundation for precision medicine in the eye.

## 2. Types and Mechanisms of Action of Small Nucleic Acid Therapeutics

### 2.1. Antisense Oligonucleotides

**Figure 1:**
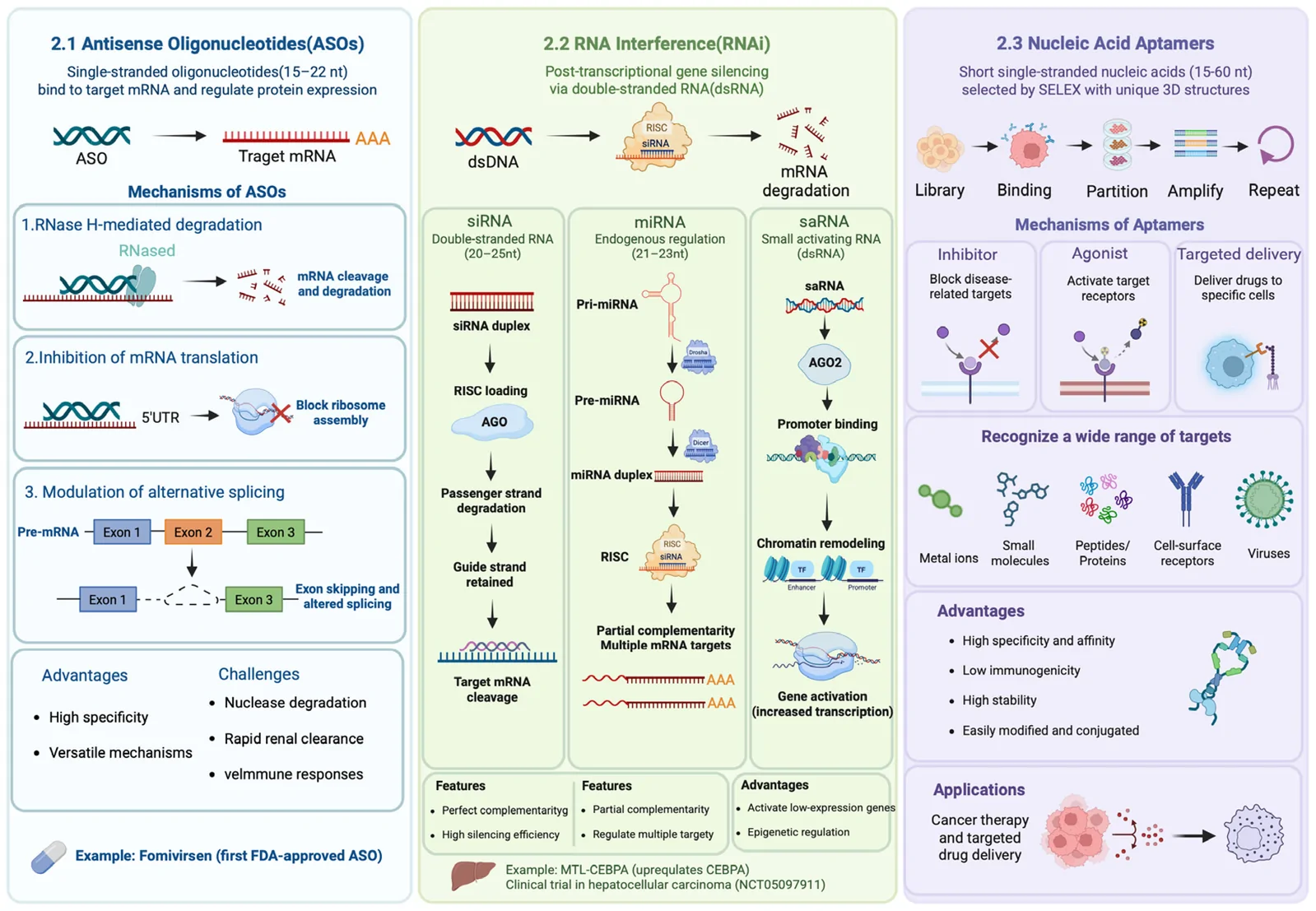
Mechanisms of action of small nucleic acid therapeutics. Created in BioRender. Zhang, H. (2026) https://app.biorender.com/citation/6aa9f3ca5bc9626b52ae3a3e (accessed on 25 August 2026).

Antisense oligonucleotides (ASOs) are single-stranded nucleic acid sequences of 15–22 nucleotides that bind to specific target mRNA sequences through Watson–Crick base pairing, thereby regulating protein expression (Figure 1). The principal mechanisms of ASOs include: (1) induction of gene silencing, in which ASOs hybridize with target RNA to form duplex structures that activate endogenous RNase H, promoting selective degradation of the target mRNA; (2) inhibition of mRNA translation, achieved by binding to ribosome recognition regions such as the 5′ untranslated region (5′-UTR), generating steric hindrance that prevents ribosomal assembly; and (3) modulation of alternative splicing, in which ASOs targeting exon-intron junctions in pre-mRNA obstruct splice-site recognition, leading to exon skipping and altered gene expression patterns [18,19,20].

Fomivirsen, an intravitreally administered ASO for cytomegalovirus retinitis, was the first approved ASO and remains historically important in ophthalmology [9]. With continued advances in oligonucleotide engineering and delivery technologies, multiple ASO-based therapeutics have since been developed for inherited retinal disorders, offering diverse strategies for clinical intervention [12]. Nevertheless, the clinical translation of ASOs remains substantially constrained by their susceptibility to nuclease-mediated degradation, rapid renal clearance, and potential for eliciting undesirable immune responses. Extensive efforts have therefore focused on developing chemical modification strategies and advanced delivery systems to prolong ASO half-life, enhance target-binding affinity, and improve overall therapeutic efficacy.

ASOs act via RNase H-mediated mRNA degradation, translational steric blockade, or alternative splicing modulation. RNAi molecules include siRNA (RISC-driven mRNA cleavage), miRNA (partial complementary binding to repress translation), and saRNA (promoter-targeted gene activation). Nucleic acid aptamers use three-dimensional structures to bind target proteins or ligands, enabling inhibition, agonism, or targeted delivery. The above image was created in BioRender. Zhang, H. (2026) https://app.biorender.com/citation/6aa9f3ca5bc9626b52ae3a3e (accessed on 25 August 2026).

### 2.2. RNA Interference

RNA interference (RNAi) is a post-transcriptional gene-silencing mechanism that selectively degrades target mRNA through the introduction of double-stranded RNA (dsRNA), thereby suppressing the expression of corresponding genes [21]. The principal classes of RNAi-related molecules include small interfering RNA (siRNA), microRNA (miRNA), and small activating RNA (saRNA) [22].

#### 2.2.1. siRNA

Small interfering RNAs (siRNAs) are double-stranded RNA molecules approximately 20–25 base pairs in length that mediate sequence-specific gene silencing [23]. Upon cellular entry, siRNAs associate with Argonaute (AGO) proteins to form the RNA-induced silencing complex (RISC). Within the RISC, the siRNA duplex undergoes unwinding, during which the passenger (sense) strand is degraded, while the guide (antisense) strand is retained to activate the complex. The activated RISC subsequently directs the antisense strand to specifically recognize complementary target mRNA, resulting in precise mRNA cleavage and degradation, thereby inhibiting translation of the corresponding gene [24]. Owing to its remarkable specificity and silencing efficiency, siRNA-mediated RNA interference enables precise post-transcriptional regulation of gene expression and has emerged as a promising therapeutic strategy for a wide range of diseases.

#### 2.2.2. miRNA

Similarly to siRNA, miRNA functions through the regulation of gene expression via partial complementary binding to target mRNAs. miRNAs are generally composed of 21–23 nucleotides and exert their regulatory effects at the post-transcriptional level [25]. The transcription of miRNAs generates primary miRNAs characterized by hairpin structures, which are subsequently cleaved by the Drosha enzyme to produce precursor miRNAs (pre-miRNAs). In the cytoplasm, pre-miRNAs undergo further processing by Dicer, resulting in the formation of mature miRNAs that are subsequently incorporated into the RNA-induced silencing complex (RISC). The principal distinction between miRNA and siRNA lies in the degree of complementarity with their target mRNAs. miRNAs typically recognize and regulate multiple target mRNAs through partial complementary base pairing, whereas siRNAs require near-perfect complementarity with target mRNAs to mediate efficient cleavage and degradation [24,26].

The development of miRNA-based therapeutics primarily encompasses two major categories: miRNA mimics and miRNA inhibitors [27]. miRNA mimics are synthetic double-stranded RNA (dsRNA) molecules designed to emulate the function of endogenous miRNAs, whereas miRNA inhibitors are single-stranded RNAs complementary to endogenous miRNAs that enhance target gene expression through specific suppression of miRNA activity. Compared with other small nucleic acid therapeutics, the development of miRNA-based drugs has progressed relatively slowly. Nevertheless, their remarkable efficacy in the treatment of cancer, heart failure, and diabetes highlights their considerable potential for clinical application, as exemplified by MRG-106, which targets miR-155 to inhibit tumor progression [28], and MRG-110, which targets miR-92 to promote angiogenesis [29]. Several ocular miRNAs are biologically compelling. miR-204 has been linked to ocular surface homeostasis and anti-inflammatory effects, including exosome-mediated dry eye therapy. miR-205 can simultaneously regulate VEGFA and ANGPT2 and is therefore relevant to pathological neovascularization, a principle consistent with the broader role of miRNA networks in regulating angiogenic signaling [30]. And miR-29 regulates extracellular matrix remodeling. In future clinical development, miRNA therapeutics will require careful disease context selection, robust safety profiling, and delivery systems that restrict activity to intended ocular cell populations.

#### 2.2.3. saRNA

Beyond the classical mechanism of gene silencing, the discovery of RNA activation has provided a novel perspective on gene regulation. Small activating RNAs (saRNAs) are a class of double-stranded RNA molecules that activate gene transcription by binding to complementary sequences within the promoter regions of target genes. This interaction facilitates the recruitment of AGO2 proteins and chromatin-modifying complexes, such as histone acetyltransferases, thereby inducing local chromatin remodeling and promoting the recruitment of RNA polymerase II [31,32]. Owing to this unique epigenetic regulatory mechanism, saRNAs offer distinct advantages in upregulating genes with low basal expression. MTL-CEBPA, developed by MiNA Therapeutics, is an saRNA therapeutic designed to target and enhance the expression of CCAAT/enhancer-binding protein alpha (CEBPA). Results from a Phase I clinical trial (NCT05097911) demonstrated that MTL-CEBPA exhibited a favorable safety profile in the treatment of hepatocellular carcinoma and may enhance the therapeutic efficacy of tyrosine kinase inhibitors through modulation of immunosuppressive pathways. In addition, several other saRNA-based therapeutic candidates are currently undergoing preclinical development for the treatment of cancer, metabolic disorders, and genetic diseases.

### 2.3. Nucleic Acid Aptamers

Nucleic acid aptamers are a class of functional nucleic acids, typically consisting of 15–60 nucleotides, that are identified through the Systematic Evolution of Ligands by Exponential Enrichment (SELEX) technique [33]. These molecules possess the ability to specifically recognize and bind target molecules. Unlike other nucleic acid therapeutics, the mechanism of action of aptamers primarily depends on the interaction between their unique three-dimensional structures and target ligands, rather than on complementary base pairing [34]. Nucleic acid aptamers can function as inhibitors by blocking disease-associated targets, as agonists by activating target receptors, or as targeted molecular carriers for delivering therapeutic agents to specific cells or tissues. Aptamers are capable of interacting with a wide range of targets, including metal ions, small-molecule compounds, peptides, proteins, cell-surface receptors, and viruses. They also exhibit several advantageous properties, such as low immunogenicity and high stability, and can be conjugated with various drugs and delivery vehicles to construct targeted drug delivery systems for cancer therapy [35]. Furthermore, researchers have introduced chemical modifications to nucleotide bases in order to confer greater structural diversity and enhanced target-binding affinity to aptamers [36].

Unlike other nucleic acid therapeutics that rely on base pairing, aptamers function through three-dimensional structure-mediated protein binding, offering extracellular action and synthetic antibody-like properties. As shown in Table 1, this class has achieved notable ocular success with pegaptanib and avacincaptad pegol, though it must compete with highly potent antibodies in clinical practice.

## 3. Chemical Modifications and Delivery Platforms

Unmodified small nucleic acids exhibit short half-lives in circulation and are highly susceptible to degradation by nucleases. Their anionic nature further hinders efficient translocation across the cell membrane, thereby limiting intracellular delivery. In addition, unmodified small nucleic acids may elicit undesirable immune responses in therapeutic applications. Consequently, it is necessary to develop chemical modification strategies and delivery systems for small nucleic acid therapeutics to enhance resistance to enzymatic degradation, ensure sequence stability, and reduce immunogenicity [32].

### 3.1. Backbone and Sugar Modifications

#### 3.1.1. Phosphate Group Modification

Phosphate backbone modification is one of the most fundamental chemical modifications. It typically involves replacing the non-bridging oxygen atoms within the phosphate backbone with alternative functional groups, such as phosphorothioate (PS), methyl phosphonate, and boranophosphate analogs [37]. PS modification is the most widely employed backbone modification strategy. By substituting oxygen atoms with sulfur atoms, it enhances the resistance of nucleic acids to nucleases, which in turn improves their stability in the bloodstream. This modification also reduces renal clearance, ultimately extending the systemic circulation time of the therapeutic agent in vivo [38]. However, although PS modification improves stability, it may also induce inflammatory responses and hepatotoxicity. For instance, Fomivirsen, a representative PS-modified drug [39], was withdrawn from the market; although its PS chemistry was associated with inflammatory effects, the withdrawal was driven primarily by the sharp decline in the incidence of cytomegalovirus retinitis following the introduction of effective antiretroviral therapy, which markedly reduced clinical demand. This has prompted the exploration of alternative modification strategies aimed at mitigating the adverse effects associated with PS chemistry [14]. Notably, combining phosphorothioate linkage with 2′ sugar modifications has enabled siRNAs to achieve robust gene-silencing activity with prolonged functional duration in vivo [40].

#### 3.1.2. Base Modification

Nucleobases are essential components of nucleic acids, and alterations to their structure can significantly affect stability, biological activity, and immunogenicity. By modifying specific sites on nucleobases, the stability and binding affinity of nucleic acids can be substantially enhanced [41,42]. Common nucleobase modifications include 5-methylcytidine (m5C), 5-fluorouracil (5-FU), N7-methylguanosine (m7G), pseudouridine, and 2′-deoxy-2′-fluorouridine (2′-FU), among others [43,44]. Yoshida et al. demonstrated that nucleobase modifications can markedly reduce the hepatotoxicity of ASOs. In addition, modifications such as m5C and pseudouridine (Ψ) enable mRNA to evade immune system recognition. Therefore, chemical modifications at these specific positions can enhance the binding affinity of nucleic acid therapeutics.

#### 3.1.3. Ribose Modification

Ribose modification represents another widely adopted strategy for optimizing oligonucleotide therapeutics through chemical modification at the 2′ position of the ribose sugar. Representative modifications include 2′-fluoro (2′-F), 2′-O-methoxyethyl (2′-MOE), and 2′-O-methyl (2′-OMe) [44,45,46,47]. These modifications improve nuclease resistance, prolong systemic circulation, and effectively reduce PS-related inflammatory responses, yielding improved safety and biological activity. Additional modifications involve simultaneous chemical alterations at the 2′ position and other ribose sites, including locked nucleic acids (LNAs) and phosphorodiamidate morpholino oligomers (PMOs). LNAs enhance oligonucleotide affinity for target sequences and increase nuclease resistance by introducing a C3′-endo conformational constraint; however, their reliance on short-sequence design can produce off-target effects and cytotoxicity. Alternative strategies have therefore been progressively adopted, including unlocked nucleic acids (UNAs), constrained ethyl-bridged nucleic acids (cEt-BNA), tricyclo-DNA (tcDNA), and glycol nucleic acids (GNAs). PMOs and peptide nucleic acids (PNAs) achieve backbone re-engineering independent of the ribose scaffold, preserving base-pairing capability while markedly improving molecular stability and target specificity. PMO-based ASOs have demonstrated clinical efficacy, most notably eteplirsen for Duchenne muscular dystrophy, validating this backbone chemistry in patients [48].

### 3.2. Delivery Systems for Oligonucleotide Therapeutics

Chemical modifications can improve the stability, target-binding affinity, and delivery properties of nucleic acid molecules. However, the inherent negative charge and hydrophilicity of nucleic acid therapeutics result in poor cellular permeability, making it difficult for them to cross biological membranes and exert their biological effects. Therefore, the selection of appropriate delivery vectors and the development of efficient delivery technologies are critical for nucleic acid-based therapies. In recent years, researchers have employed lipid-based systems, polymeric carriers, inorganic nanomaterials, and exosomes as delivery vehicles for nucleic acid therapeutics, thereby enhancing drug stability and biocompatibility [49].

#### 3.2.1. Lipid Nanoparticles

Lipid nanoparticles (LNPs) are spherical, solid nanoparticles composed of four key lipid components: ionizable cationic phospholipids, neutral helper phospholipids, cholesterol, and polyethylene glycol (PEG)-modified phospholipids. Nucleic acid therapeutics encapsulated within LNPs are protected from nuclease-mediated degradation and efficiently delivered to target tissues. The lipophilic nature of LNPs facilitates fusion with cellular membranes, promoting intracellular cargo delivery. LNPs also reduce recognition by Toll-like receptors, thereby minimizing innate immune overactivation. Patisiran, an siRNA therapeutic delivered via an LNP system, received approval in 2018, representing the first clinical application of LNP technology and the first marketed non-viral gene delivery system [50]. More recently, siRNA-LNP systems targeting highly conserved regions of the SARS-CoV-2 genome have achieved viral inhibition rates exceeding 90%, demonstrating strong antiviral potential as adjuncts to vaccination strategies [51]. In ophthalmology, LNPs form stable interactions with corneal and conjunctival surfaces, enhancing ocular drug absorption. LNP formulations have successfully delivered mRNA to retinal pigment epithelial cells and Müller glial cells, significantly improving therapeutic outcomes while enabling controlled drug release.

#### 3.2.2. N-Acetylgalactosamine (GalNAc)

GalNAc-modified RNA systems represent an efficient, liver-targeted delivery technology that has become one of the most widely used nucleic acid delivery platforms [52]. GalNAc serves as a high-affinity ligand for the asialoglycoprotein receptor (ASGPR), which is highly and selectively expressed on hepatocyte surfaces. ASGPR-mediated, clathrin-dependent endocytosis efficiently transports GalNAc conjugates from the cell surface into the cytoplasm. Following subcutaneous administration, GalNAc-conjugated therapeutics rapidly accumulate in the liver, are internalized by hepatocytes through ASGPR-mediated uptake, and are gradually released from endosomes, enabling sustained gene-silencing effects [53,54]. This platform has achieved substantial clinical success in treating liver diseases, with representative agents including Alnylam’s Givlaari (acute hepatic porphyria), inclisiran (hypercholesterolemia), and lumasiran (primary hyperoxaluria) [55]. Researchers have also developed GalNAc analogs, such as chemically modified mannose conjugates, to enable siRNA delivery to macrophages and dendritic cells via CD206 binding, opening new avenues for extrahepatic applications of GalNAc-based delivery [56]. Because ASGPR expression is essentially confined to hepatocytes, unmodified GalNAc conjugates have little relevance for ocular tissues; ocular applications of sugar-based targeting therefore depend on alternative receptors or on local intraocular administration, where ligand-mediated uptake is not required.

#### 3.2.3. Polymeric Nanocarriers

Polymeric nanoparticles are fabricated from natural polymers such as dextran, chitosan, and cyclodextrin, or from synthetic polymers, enabling nanomaterials with diverse compositions and architectures [57]. Common forms include nanocapsules and nanospheres, further categorized into polymers, polymeric assemblies, and dendrimers. Polymeric nanoparticles offer straightforward synthesis, structural diversity, scalable production, high transfection efficiency, low immunogenicity, and good biocompatibility, making them among the most promising nanocarriers for nucleic acid delivery. PEGylated lipid–protein–hyaluronic acid nanoparticles (PEG-LPH-NP-S) have been shown to effectively reduce CNV in a mouse model [58]. In addition, octopus-like multivalent cell-penetrating peptides modified with polyethylene glycol have demonstrated efficient delivery of therapeutic siRNA, highlighting strong potential for non-invasive intraocular gene delivery [59].

#### 3.2.4. Inorganic Nanocarriers

Inorganic nanomaterials, including gold nanoparticles (AuNPs), silicon dioxide nanoparticles (SiO_2_ NPs), and iron oxide nanoparticles (Fe_3_O_4_ NPs), have attracted extensive attention as carriers for nucleic acid delivery and bioimaging [60]. These nanoparticles can be engineered with precise control over size, structure, and geometry. Gold and iron oxide nanoparticles are generally considered relatively non-toxic. Hybrid inorganic–organic nanocarriers have demonstrated strong potential for targeted delivery and multifunctional applications [61]. Stimulus-responsive silica nanoparticles have been developed to efficiently deliver plasmid DNA, mRNA, and CRISPR-Cas9 gene-editing components into retinal pigment epithelial (RPE) cells and hepatocytes in mice, enabling effective genome modification [62,63]. For retinoblastoma treatment, polyethyleneimine-modified gold nanoparticles conjugated with epithelial cell adhesion molecule (EpCAM) antibodies achieve active EpCAM-mediated targeting, delivering siRNA into tumor cells and significantly inhibiting tumor proliferation [64]. These hybrid nanocarriers, by virtue of their unique electrical, magnetic, and optical properties, further expand the scope of RNA delivery applications.

#### 3.2.5. Exosomes

Exosomes are nanoscale vesicles (approximately 30–150 nm in diameter) actively secreted by cells into the extracellular space [65,66,67]. They carry a rich repertoire of bioactive molecules, including proteins, nucleic acids (mRNA, miRNA, lncRNA), lipids, and metabolites, which are delivered to target cells via paracrine signaling or the circulatory system, thereby regulating physiological and pathological processes in recipient cells. As natural nanocarriers, exosomes have emerged as a promising platform for nucleic acid drug delivery, capable of encapsulating small molecules, nucleic acid drugs, recombinant proteins, and CRISPR/Cas9 systems [68,69,70]. Exosomal membranes express specific ligands and adhesion molecules that mediate interactions with recipient cell membranes or facilitate cellular uptake, enabling intracellular release of therapeutic payloads. Compared with liposomes and other polymer-based nanocarriers, exosomes contain transmembrane and membrane-anchored proteins that enhance endocytosis and promote more efficient drug delivery [71]. Tumor-derived exosomes express CD47 on their surface, which binds to signal regulatory protein alpha (SIRPα) on macrophages, enabling immune evasion and improving stability in the biological milieu [72]. Exosomes from different cellular sources exhibit distinct functional properties: HEK293-derived exosomes are immunologically inert; cancer cell-derived exosomes overexpressing Rab27a and Rab27b inherit functional characteristics of their parental cells; and exosomes secreted by monocytes and macrophages possess the ability to evade phagocytic clearance [73].

**Figure 2:**
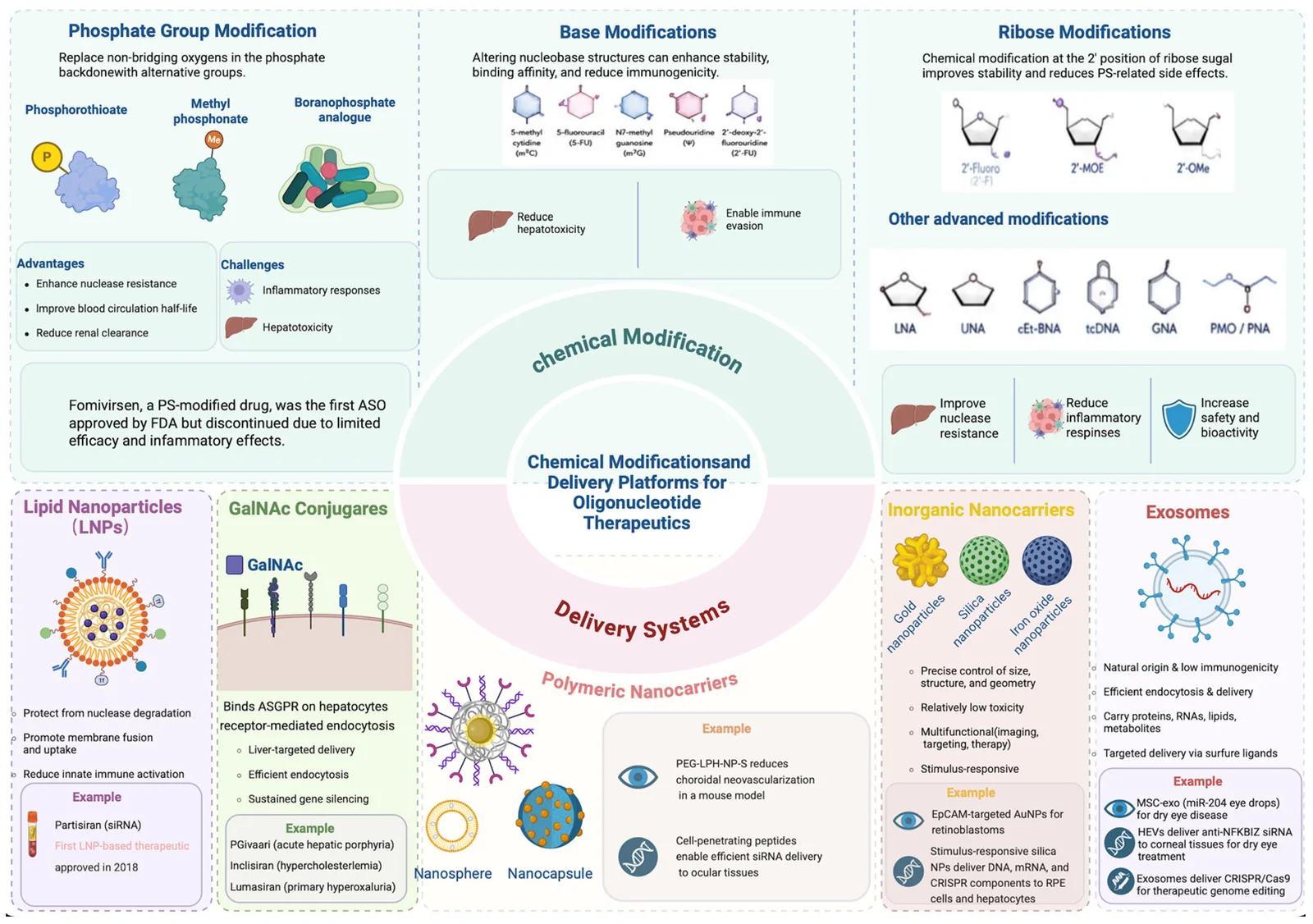
Chemical modifications and delivery systems for small nucleic acid therapeutics.

Numerous studies have explored exosome-mediated nucleic acid delivery. Exosomes can effectively deliver ASOs, siRNAs, and miRNAs with promising anti-tumor efficacy. One group developed a mesenchymal stem cell-derived exosome-based eye drop (MSC-exo) loaded with miR-204, providing a safe, non-invasive therapeutic strategy for dry eye disease [74]. In another study, a DNA zipper-mediated membrane fusion approach was used to construct hybrid exosome vectors (HEVs), in which liposomes encapsulating anti-NFKBIZ siRNA were fused with corneal epithelial cell-derived exosomes to generate HEVs for dry eye treatment. Owing to the homing properties inherited from their parent exosomes, siRNA-loaded HEVs specifically target corneal tissues and efficiently deliver their cargo, reshaping the ocular surface inflammatory microenvironment toward a physiological state [75]. With the rapid development of CRISPR gene-editing technologies, exosomes have also been used to deliver CRISPR/Cas9 systems for therapeutic genome editing. Dozens of companies worldwide have now developed exosome-based therapeutic platforms. Exosomes hold broad prospects as both diagnostic tools and nucleic acid delivery vehicles; however, compared with LNPs, exosome-based products still face unresolved challenges in scalable GMP manufacturing, batch-to-batch reproducibility, and defined formulation control, which currently place them at an earlier translational stage than LNP platforms (Figure 2).

Upper panel: Backbone modifications, base modifications, and ribose modifications (e.g., 2′-F, 2′-MOE, 2′-OMe, LNA, PMO, PNA). Lower panel: Delivery systems including lipid nanoparticles (LNPs), GalNAc conjugates, polymeric nanocarriers, inorganic nanocarriers, and exosomes. The above image was created in BioRender. Zhang, H. (2026) https://app.biorender.com/citation/6a8c263e00a9828936fc1df8 (accessed on 25 August 2026).

#### 3.2.6. Delivery Routes for Ocular Nucleic Acid Therapeutics

Beyond the choice of carrier, the route of administration determines which ocular tissues can be reached and which modalities are feasible. Topical instillation is non-invasive, but the tear film, corneal epithelium, and conjunctival clearance restrict access to the ocular surface and anterior segment, and only a small fraction of an instilled dose reaches intraocular tissues; it is therefore suitable mainly for surface disease, as exemplified by tivanisiran. Intravitreal injection delivers high vitreous concentrations to the inner retina and RPE, making it the standard route for ASOs, siRNAs, and aptamers in retinal disease, at the cost of repeated invasive procedures and their risks. Subretinal injection provides direct access to photoreceptors and RPE and is the route of choice for gene-replacement vectors in IRD, but it requires vitrectomy and treats a limited retinal area. Suprachoroidal injection, typically via microneedle, targets the choroid and outer retina with minimal surgical trauma, and has recently re-emerged as a promising route for nucleic acid and gene therapies, although clinical experience remains limited. Table 2 summarizes these routes in terms of accessible tissues, suitable modalities, durability, invasiveness, and key safety considerations.

## 4. Therapeutic Applications in Ocular Diseases

### 4.1. Age-Related Macular Degeneration

AMD is a leading cause of irreversible vision loss in the elderly, classified into dry AMD (dAMD) and wet AMD (wAMD). Dry AMD, the more common form, is characterized in its early and intermediate stages by drusen accumulation, RPE abnormalities, and Bruch’s membrane thickening. Advanced stages involve focal RPE degeneration and photoreceptor loss, a condition termed geographic atrophy (GA). Some cases progress further to choroidal neovascularization (CNV), culminating in irreversible vision impairment. Therapeutic options for dry AMD remain limited [76]. Recently, Iveric Bio (acquired by Astellas Pharma in 2023) developed an intravitreally administered RNA aptamer targeting complement component C5, addressing one of the key pathogenic mechanisms of dAMD involving the complement system. Avacincaptad pegol (ACP), a C5-targeting aptamer, received U.S. FDA approval in 2023 for the treatment of geographic atrophy secondary to AMD [77]. The key innovation of ACP lies in its RNA aptamer structure, which enables high-affinity, highly specific C5 binding, blocking cleavage into C5a and C5b-9 and thereby inhibiting terminal complement pathway overactivation [78]. As the first approved RNA aptamer therapy for dAMD-associated GA, ACP represents a significant therapeutic advance [79]. Future efforts should focus on non-invasive delivery routes and long-acting delivery systems to provide safer, more durable treatment.

Wet AMD is characterized primarily by CNV formation, largely driven by excessive activation of VEGF and related signaling pathways. Therapeutics targeting VEGF have therefore become the mainstay of wAMD treatment. Oligonucleotide therapeutics, by virtue of their ability to precisely regulate gene expression, have shown unique potential in this space. Bevasiranib and Sirna-027 are siRNA therapeutics targeting VEGF-A and VEGFR1, respectively. Both showed signs of biological activity in early-phase testing [80], but neither translated into durable clinical benefit: bevasiranib’s Phase III program was terminated for futility, and the Phase II study of Sirna-027 (AGN-745) failed to meet its primary endpoint [81]. These outcomes are generally attributed to inadequate intracellular delivery, rapid intravitreal clearance, and pharmacodynamic durability too short to compete with anti-VEGF biologics. To overcome these limitations, OliX Pharmaceuticals developed a novel RNAi-based gene silencing platform known as asymmetric siRNA (asiRNA) [82]. OLX10212, a cell-penetrating asiRNA targeting MyD88, a central adaptor protein upstream of inflammasome-driven angiogenesis, inhibits neovascularization (NCT05643118). Compared with conventional siRNA, asiRNA not only provides comparable gene-silencing efficiency but also significantly reduces siRNA-associated adverse effects such as off-target gene silencing and immune stimulation. Phase I testing (NCT05643118) demonstrated favorable safety and preliminary improvements in best-corrected visual acuity (BCVA), and a Phase IIa study in geographic atrophy is in preparation. Historically, pegaptanib (Macugen), a VEGF165-targeting RNA aptamer, was the first ocular aptamer approved (2004) for neovascular AMD. Although later displaced by pan-VEGF biologics, it remains a landmark proof of concept for RNA-based therapies in the retina (Table 3). Beyond VEGF-targeted therapies, the RNA aptamer RBM-007 targets fibroblast growth factor 2 (FGF2) and exhibits dual anti-angiogenic and anti-fibrotic activity; its Phase II trials (TOFU/SUSHI) have been completed with published results [83]; no Phase III program has been initiated to date. ASOs targeting transforming growth factor-β2 (TGF-β2) have also been developed to inhibit both neovascularization and retinal fibrosis [84]. Collectively, these agents hold promise as adjunctive or alternative therapies to anti-VEGF treatment in neovascular AMD.

### 4.2. Glaucoma

Glaucoma is characterized by irreversible optic nerve damage and visual field loss resulting from pathological elevation of intraocular pressure (IOP). Current clinical management focuses on IOP reduction through pharmacological interventions and surgical procedures such as trabeculectomy. However, the long-term success of filtering surgery is limited by postoperative excessive fibrotic proliferation, which can lead to drainage pathway closure and suboptimal IOP control [85,86]. Although existing therapies can delay disease progression, they do not address core pathological mechanisms such as retinal ganglion cell (RGC) apoptosis, optic nerve axonal injury, and neuroinflammation. There is therefore a critical need for novel strategies targeting neuroprotection, extracellular matrix remodeling, and fibrotic signaling pathways including TGF-β and Wnt/β-catenin.

QPI-1007 (cosdosiran) is a chemically modified siRNA that reduces RGC apoptosis by targeting caspase-2 mRNA. It was evaluated in a Phase II study in glaucoma (NCT01965106), and a large randomized, sham-controlled Phase II/III trial in acute non-arteritic anterior ischemic optic neuropathy (NAION; NCT02341560) did not meet its primary endpoint at six months; post hoc analyses nevertheless suggested that patients with more severe baseline visual loss derived measurable benefit [87]. ISTH0036, an ASO selectively targeting human TGF-β2, reduces tissue fibrosis and contributes to IOP lowering; it has received orphan drug designation from the U.S. FDA for this indication and is in clinical development, but it is not an approved marketed therapy. A first-in-human Phase I study confirmed a favorable safety profile and early anti-fibrotic signals: among the 12 post-surgical glaucoma patients treated with ISTH0036, 75% achieved the pre-specified IOP criterion at 3 months [88]. Although encouraging, these findings derive from a very small, open-label sample (*n* = 12) and require confirmation in larger controlled trials. SYL040012, an siRNA targeting the β2-adrenergic receptor (ADRB2) pathway, demonstrated IOP-lowering effects in Phase I trials with a favorable local safety profile and without the systemic β-blockade effects associated with timolol [89]; no active late-phase development has been reported. These studies collectively demonstrate the unique advantages of oligonucleotide therapeutics in glaucoma. Future efforts should focus on optimizing delivery systems such as nanocarriers or cell-penetrating peptide modifications to enhance intraocular targeting and bioavailability, and on exploring combination strategies such as ASO therapy with neurotrophic factors to synergistically promote RGC survival and visual function recovery.

### 4.3. Diabetic Retinopathy

DR is among the most prevalent microvascular complications of diabetes, characterized by blood–retinal barrier disruption, inflammatory mediator release, pathological neovascularization, and glial cell activation, ultimately culminating in irreversible visual impairment [90,91,92]. Current pharmacological management relies predominantly on anti-VEGF antibody-based therapies. Although these agents have demonstrated considerable efficacy, their clinical utility is constrained by the need for frequent intravitreal injections. In addition, there is a propensity for resistance arising from their single-target mechanisms of action [93].

ISTH0036, an ASO targeting TGF-β2 through a non-VEGF pathway, has shown efficacy in DR beyond its potential in neovascular AMD and glaucoma. Preliminary results of the Phase II BETTER trial indicate that intravitreal ISTH0036 reduced central retinal thickness and hyperreflective foci in diabetic macular edema (DME) by suppressing fibrosis and angiogenesis; notably, 71% of DME patients reduced the frequency of anti-VEGF treatments [84]. These findings require confirmation in larger, longer studies. ICO-007 is a second-generation ASO that targets c-Raf kinase, a shared downstream signaling node of multiple pro-angiogenic and permeability-inducing growth factors, including VEGF, IGF, bFGF, EPO, and HGF. By inhibiting pathological angiogenesis and preventing blood–retinal barrier disruption, ICO-007 may overcome the limitations of single-target interventions [94]. Phase I testing in DME patients demonstrated a favorable safety profile; however, no active late-phase development has been reported. Future investigations should focus on validating long-term safety and therapeutic superiority through Phase II/III trials. In addition, they should explore the synergistic potential of advanced delivery systems and multimodal therapeutic approaches, moving DR treatment toward a paradigm of “single injection, multifaceted therapeutic benefit.”

### 4.4. Inherited Retinal Diseases

Inherited retinal diseases (IRDs) comprise a heterogeneous group of retinal dysfunction disorders caused by monogenic or polygenic mutations [95]. IRDs can be broadly categorized into disorders primarily characterized by functional abnormalities of photoreceptors or bipolar cells without significant cellular degeneration. They also include disorders marked by progressive photoreceptor apoptosis and structural retinal abnormalities, ultimately resulting in irreversible vision loss [95]. The global prevalence is estimated at approximately 1 in 2000 individuals. Owing to their high propensity for blindness and frequent onset in younger populations, IRDs represent a major challenge in visual health.

Several siRNA- and ASO-based therapeutics for IRDs are undergoing clinical evaluation with encouraging outcomes. Sepofarsen (QR-110) is an ASO developed for the treatment of Leber congenital amaurosis type 10 (LCA10) caused by the p.Cys998X mutation in the centrosomal protein 290 (CEP290) gene. In a Phase II/III ILLUMINATE trial (NCT03140969), sepofarsen did not meet its primary endpoint (best-corrected visual acuity at 12 months); nevertheless, early-phase results demonstrated improved retinal sensitivity (full-field stimulus testing) in most treated eyes [96], and durable vision improvement has been documented in individual patients [97]. The program was subsequently acquired by Laboratoires Théa and continues in clinical development under Sepul Bio. The ASO QR-421a (ultevursen), which induces skipping of exon 13 of USH2A, improved retinal sensitivity in early-phase studies in retinitis pigmentosa and Usher syndrome patients [98]. Its Phase II/III SIRIUS trial was terminated by the original sponsor, and development has since been resumed under Sepul Bio (Laboratoires Théa), with a Phase IIb trial (LUNA) now underway. Researchers have also developed ASOs targeting the 5′ uORF, exon 3, exon 11, and exon 14 regions of PRPF31 pre-mRNA, effectively enhancing PRPF31 gene expression and providing a novel therapeutic strategy for retinitis pigmentosa [99,100]. Collectively, these advances substantiate the feasibility of small nucleic acid drugs in ophthalmic gene therapy. The design of mutation-specific therapeutics tailored to distinct genetic abnormalities such as splicing defects and nonsense mutations holds substantial promise for advancing precision and personalized treatment of IRDs.

### 4.5. Dry Eye Disease

Dry eye disease (DED) is a prevalent ocular disorder characterized by tear film instability, ocular inflammation, and neurosensory abnormalities. Clinically, DED is broadly classified into aqueous-deficient and evaporative subtypes. Current treatment for mild-to-moderate DED relies primarily on artificial tears for ocular surface lubrication and symptom relief, whereas severe cases often require autologous serum eye drops or anti-inflammatory medications [101]. Existing treatment modalities remain limited, particularly for moderate-to-severe disease. There is an urgent need for innovative approaches capable of both relieving symptoms and modifying disease progression across diverse etiological subgroups. Because tear turnover clears topical agents within minutes, the short ocular surface residence time that limits topical nucleic acid delivery is shared by all topical DED therapies; non-nucleic-acid strategies that address the same barrier illustrate alternative formulation routes. For example, an injectable in situ-forming copolymer that continuously releases a lubricating component on the ocular surface effectively relieved signs and symptoms of DED in preclinical models, highlighting the value of residence-time engineering [102]. Nucleic acid-based DED therapies will need to solve the same problem, for instance through mucoadhesive formulations, sustained-release matrices, or exosome-mediated carriers.

Tivanisiran (SYL1001) is an siRNA-based ophthalmic formulation designed to silence transient receptor potential vanilloid 1 (TRPV1) mRNA, thereby alleviating ocular discomfort and pain [103,104]. In the Phase III HELIX trial (NCT03108664), tivanisiran did not meet its co-primary endpoints (ocular pain and total corneal staining) versus artificial tears in the overall population, but significantly reduced central corneal staining and showed consistent signals of benefit in the Sjögren’s syndrome subgroup, with a favorable safety profile.

## 5. Discussion and Perspectives

Small nucleic acid therapeutics have become an important strategy in ophthalmic precision medicine. Their significance lies not only in providing a new drug class, but also in shifting therapeutic intervention upstream to the RNA level. Through base pairing or structure-dependent recognition, these agents can degrade pathogenic transcripts, alter splicing, restore regulatory RNA activity, activate selected genes, or block extracellular targets through aptamer binding [14,105]. This mode of action expands the therapeutic space for ocular diseases that are difficult to manage with conventional small-molecule drugs or protein-based therapies. It is particularly relevant across a wide spectrum of ocular diseases, from inherited retinal dystrophies to acquired vascular, inflammatory, and degenerative conditions [16].

This therapeutic potential is closely linked to the molecular complexity of ocular diseases, but its clinical value differs across disease settings. In neovascular AMD, DR, and DME, disease progression involves more than VEGF signaling alone. Inflammation, vascular leakage, fibrosis, extracellular matrix remodeling, ischemia, and neuronal injury also contribute to disease heterogeneity and incomplete treatment response. Small nucleic acid therapeutics may therefore provide opportunities to regulate pathways beyond VEGF or to modulate several disease-related mechanisms at the same time. In IRD, the rationale is more genotype-directed. Many of these disorders arise from defined mutations, which makes transcript-directed treatment biologically attractive. ASOs, siRNAs, and related approaches can be designed to correct aberrant splicing, reduce toxic transcripts, or modulate mutation-associated RNA effects [17,106]. ASO-based clinical programs targeting genes such as CEP290 (sepofarsen/QR-110) and USH2A (QR-421a) have shown the feasibility of sequence-guided intervention in retinal tissue, with measurable biological or functional effects reported in selected patients [107,108]. Together, vascular conditions and IRD illustrate the same translational principle: molecular precision must be converted into durable, anatomically relevant, and clinically meaningful benefit.

A rational target and a well-designed sequence are not sufficient on their own: the candidate must reach the correct ocular compartment, enter the relevant cell type, remain active for an adequate duration, and produce an effect that matters to patients [14,109]. Despite substantial advances in ocular nucleic acid therapeutics over the past two decades, only a limited number of programs have successfully translated into approved therapies, reflecting several persistent barriers to clinical development. These challenges include target validation, as several early VEGF-pathway siRNA programs, including bevasiranib and Sirna-027, were directed against biologically plausible but functionally redundant nodes within an axis already effectively targeted by antibody-based therapies [110]; delivery, as native or minimally modified oligonucleotides are rapidly cleared from the vitreous and exhibit limited uptake by retinal cells; pharmacodynamic durability, as transient target suppression may not achieve a therapeutic window comparable to the sustained efficacy and dosing intervals of contemporary anti-VEGF biologics; disease stage, as genotype-directed therapies have often been evaluated in patients with advanced retinal degeneration, where irreversible structural loss may restrict functional recovery despite successful molecular correction [111]; and competition with established standards of care, as emerging therapies must demonstrate meaningful clinical advantages over effective and widely adopted treatments rather than merely biological activity. Addressing these translational barriers will require coordinated advances in oligonucleotide chemistry, delivery technologies, clinical endpoint selection, and trial design.

Even with well-designed endpoints, however, clinical benefit cannot be achieved without adequate tissue exposure. Delivery remains the major barrier between molecular design and clinical impact [112,113]. The eye is suitable for local treatment, but ocular tissues are protected by several anatomical and physiological barriers. The tear film, corneal epithelium, conjunctiva, vitreous, inner limiting membrane, retinal pigment epithelium, and blood–retinal barrier all restrict drug distribution. These barriers preserve ocular homeostasis, but they also limit bioavailability and tissue access. Posterior segment diseases are especially challenging, and many current approaches still depend on intravitreal injection. This route provides high intraocular exposure, but repeated injections increase the risk of procedure-related complications such as endophthalmitis, retinal detachment, and vitreous hemorrhage. They also increase the long-term treatment burden. New delivery systems, including lipid nanoparticles, polymeric carriers, extracellular vesicles, hydrogels, microneedles, and sustained release platforms, may improve molecular protection, tissue retention, cellular uptake, or dosing interval. However, each platform introduces distinct biocompatibility and toxicity profiles that must be carefully evaluated [114]. Moreover, no single route or platform is suitable for all indications. Topical delivery is more appropriate for ocular surface and corneal diseases. Intravitreal injection remains practical for vascular and inner retinal targets. Subretinal delivery provides direct access to photoreceptors and retinal pigment epithelium [115], but it is more invasive. Suprachoroidal delivery may offer access to the choroid and outer retina with less surgical trauma. Delivery design must therefore be tailored to disease anatomy, target cell localization, payload properties, required retention time, dosing interval, and safety profile.

Molecular design and safety evaluation must be developed in parallel with delivery optimization. Native nucleic acids are rapidly degraded by nucleases and usually require chemical modification to achieve sufficient stability in ocular tissues [116]. Phosphorothioate backbone modification, 2′-O methylation, 2′-fluoro substitution, and locked nucleic acid incorporation can improve nuclease resistance, binding affinity, and pharmacokinetic behavior [117]. These modifications may also alter hybridization, protein binding, tissue distribution, immune recognition, and toxicity. The optimal design should balance stability, potency, specificity, intracellular activity, and ocular safety. This balance is essential because excessive modification may prolong half-life but reduce biological activity or increase tissue-related adverse effects. Safety assessment should extend beyond conventional toxicity testing. Partial sequence complementarity may inadvertently suppress unintended transcripts, leading to off-target effects that are difficult to predict, while certain nucleotide motifs may activate innate immune pathways [118]. Carrier materials may also induce adverse responses; for instance, certain lipid nanoparticles, particularly upon systemic administration, can trigger complement activation-related pseudoallergy (CARPA), while some polycationic carriers may cause retinal pigment epithelium stress or local inflammation. Preclinical evaluation should therefore include target engagement, transcriptome wide off-target analysis, innate immune activation, ocular biodistribution, tissue retention, and local toxicity. Computational prediction and high-throughput sequencing can improve early screening, but their results should be validated in relevant ocular cells, retinal organoids, animal models, and clinical samples.

Overall, the future success of small nucleic acid therapeutics in ophthalmology will depend on coordinated development rather than on a promising sequence alone. Target selection, oligonucleotide chemistry, delivery route, formulation design, patient stratification, and endpoint selection should be considered together from the earliest stages. Genetic testing can define mutation status [119]. Transcriptomic and proteomic profiling can reveal active disease pathways. Sensitive outcome measures, as discussed above, should be incorporated alongside molecular diagnostics and biomarker profiling to define disease stage, stratify patients, and monitor therapeutic response [111]. With continued progress in nucleic acid chemistry, ocular delivery science, molecular diagnostics, and clinical trial methodology, small nucleic acid therapeutics may provide durable and individualized treatment options for a wide spectrum of inherited and acquired ocular diseases.

## 6. Conclusions

Small nucleic acid therapeutics have emerged as a promising strategy in ophthalmology by virtue of their high target specificity and capacity to modulate disease-relevant genes. Recent advances in siRNA- and ASO-based therapies have demonstrated considerable potential in retinal vascular diseases, inherited retinal disorders, and ocular surface diseases, underscoring their value in precision medicine and personalized therapy [120]. Several challenges nevertheless continue to limit broader clinical application, including insufficient ocular delivery efficiency, molecular instability, immunogenicity, off-target effects, and long-term safety concerns. Manufacturing complexity and regulatory standardization pose additional barriers to translation. Future research should prioritize optimized sequence design, improved delivery systems, and the establishment of standardized safety and efficacy evaluation frameworks. With continued advances in nanotechnology, biomaterials, and gene regulation strategies, small nucleic acid therapeutics are poised to improve the treatment of ocular diseases and provide more precise, effective, and individualized therapeutic options for patients with vision-threatening disorders.

## Figures and Tables

**Table 1:** Small nucleic acid therapeutic classes relevant to ophthalmology.

Class	Primary Mechanism	Main Advantages	Key Limitations	Representative Ocular Examples (Development Status)
ASO	RNase H-mediated RNA degradation or steric blocking/splice modulation	Single-stranded; can target nuclear pre-mRNA; strong chemical stability	Dose-related toxicity, immune activation, sequence-dependent off-targets effects	Fomivirsen (approved 1998, withdrawn); Sepofarsen/QR-110 (Phase II/III, primary endpoint not met); ISTH0036 (Phase II); Ultevursen/QR-421a (Phase IIb)
siRNA	RISC-mediated mRNA cleavage	High potency and durable silencing with modern modifications	Requires efficient cytoplasmic delivery and endosomal escape; limited durability in early programs	Bevasiranib and Sirna-027 (discontinued); Tivanisiran (Phase III completed); OLX10212 (Phase I completed); QPI-1007 (Phase II/III completed)
miRNA mimic/inhibitor	Post-transcriptional regulation of multiple target genes	Network-level regulation angiogenesis, inflammation, fibrosis, and survival	Limited target-specific; complex safety evaluation	No approved ocular miRNA mimic/inhibitor therapies to date; ocular applications remain primarily at the preclinical stage
Aptamer	Structure-dependent protein binding	Extracellular action; antibody-like targeting properties; synthetic production	Competition with highly effective biologics; structural stability requirements	Pegaptanib; avacincaptad pegol; corneal-targeting aptamers; RBM-007 (Phase II completed)
saRNA	Promoter-targeted transcriptional activation	Activates endogenous protective genes; potential long-lasting effects	Early developmental stage; unresolved delivery and chromatin specificity issues	MTL-CEBPA (Phase I, HCC); ocular applications remain preclinical

**Table 2:** Comparison of ocular delivery routes for nucleic acid therapeutics.

Delivery Route	Accessible Tissues/Cells	Suitable Modalities	Durability	Invasiveness	Key Safety Concerns
Topical	Cornea, conjunctiva, tear film; limited anterior segment	siRNAs, ASOs, modified oligonucleotides	Hours; rapid clearance	Non-invasive	Surface toxicity; poor posterior delivery
Intravitreal	Vitreous, inner retina, partial RPE	ASOs, siRNAs, aptamers, sustained-release systems	Weeks; repeated dosing often needed	Minimally invasive	Endophthalmitis, retinal injury, IOP elevation
Subretinal	Photoreceptors, RPE	AAV therapy, nucleic acids for IRDs	Months–years (mainly gene therapy)	Surgical	Retinal detachment, limited coverage
Suprachoroidal	Choroid, outer retina, RPE	Nucleic acids, small molecules, viral vectors	Intermediate; formulation-dependent	Minimally invasive	Choroidal effusion/hemorrhage; limited experience

**Table 3:** Selected clinical or translational small nucleic acid therapeutics in ophthalmology.

Agent	Class	Target	Indication	Administration	Clinical Status	Outcome and Translational Significance
Fomivirsen	ASO	CMV IE2 mRNA	CMV retinitis	Intravitreal	Approved 1998; withdrawn	First approved ASO; withdrawal mainly due to reduced CMV retinitis incidence after antiretroviral therapy
Pegaptanib	Aptamer	VEGF165	nAMD	Intravitreal	Approved 2004; discontinued	Reduced vision loss; later displaced by broader anti-VEGF biologics
Avacincaptad pegol/Izervay	Aptamer	Complement C5	Geographic atrophy AMD	Intravitreal	Approved 2023	Slowed GA lesion growth; developed by Iveric Bio (acquired by Astellas Pharma in 2023)
Bevasiranib	siRNA	VEGF-A	nAMD	Intravitreal	Phase III terminated	Failed due to insufficient efficacy compared with established anti-VEGF therapy
Sirna-027 (AGN-745)	siRNA	VEGFR1	CNV	Intravitreal	Phase II terminated	Failed primary endpoint; highlighted durability and delivery challenges
OLX10212	Cp-asiRNA	MyD88	nAMD/GA	Intravitreal	Phase I completed	Safety established; designed to improve uptake and reduce off-target effects
ISTH0036	LNA-ASO	TGF-beta2	Glaucoma surgery/DME/AMD	Intravitreal/ subconjunctival	Phase II ongoing	FDA orphan designation; clinical development ongoing; not an approved therapy
SYL040012 (bamosiran)	siRNA	ADRB2	Glaucoma	Topical	Phase I/II	IOP reduction observed; no late-phase development reported
QPI-1007 (cosdosiran)	siRNA	Caspase-2	NAION/ glaucoma	Intravitreal	Phase II/III completed	Primary endpoint not achieved; subgroup signals observed
Sepofarsen (QR-110)	ASO	CEP290 p.Cys998X	LCA10	Intravitreal	Phase II/III completed	Primary endpoint not met; early retinal sensitivity improvement; continued development
ultevursen (QR-421a)	ASO	USH2A exon 13	RP/Usher syndrome	Intravitreal	Phase II/III terminated; Phase IIb ongoing	Early retinal sensitivity improvement; development resumed under Sepul Bio
Tivanisiran (SYL1001)	siRNA	TRPV1	Dry eye disease	Topical	Phase III completed	Overall endpoint not met; subgroup signals observed with favorable safety
RBM-007	Aptamer	FGF2	nAMD	Intravitreal	Phase II completed	Anti-angiogenic/anti-fibrotic proof of concept; no Phase III initiated
ICO-007	ASO	RAF1	dme	Intravitreal	Phase I completed	Favorable safety profile; no late-phase development

## Data Availability

No new data were created or analyzed in this study. Data sharing is not applicable to this article.
